# Anti-Inflammatory and Immunomodulatory Effects of Probiotics in Gut Inflammation: A Door to the Body

**DOI:** 10.3389/fimmu.2021.578386

**Published:** 2021-02-26

**Authors:** Fernanda Cristofori, Vanessa Nadia Dargenio, Costantino Dargenio, Vito Leonardo Miniello, Michele Barone, Ruggiero Francavilla

**Affiliations:** ^1^Department of Biomedical Science and Human Oncology, University of Bari Aldo Moro, Bari, Italy; ^2^Gastroenterology Unit, Department of Emergency and Organ Transplantation, University of Bari Aldo Moro, Bari, Italy

**Keywords:** probiotics, microbiota, inflammation, celiac disease, inflammatory bowel disease, irritable bowel syndrome, obesity, autism spectrum disorders

## Abstract

Hosting millions of microorganisms, the digestive tract is the primary and most important part of bacterial colonization. On one side, in cases of opportunistic invasion, the abundant bacterial population inside intestinal tissues may face potential health problems such as inflammation and infections. Therefore, the immune system has evolved to sustain the host–microbiota symbiotic relationship. On the other hand, to maintain host immune homeostasis, the intestinal microflora often exerts an immunoregulatory function that cannot be ignored. A field of great interest is the association of either microbiota or probiotics with the immune system concerning clinical uses. This microbial community regulates some of the host’s metabolic and physiological functions and drives early-life immune system maturation, contributing to their homeostasis throughout life. Changes in gut microbiota can occur through modification in function, composition (dysbiosis), or microbiota–host interplays. Studies on animals and humans show that probiotics can have a pivotal effect on the modulation of immune and inflammatory mechanisms; however, the precise mechanisms have not yet been well defined. Diet, age, BMI (body mass index), medications, and stress may confound the benefits of probiotic intake. In addition to host gut functions (permeability and physiology), all these agents have profound implications for the gut microbiome composition. The use of probiotics could improve the gut microbial population, increase mucus-secretion, and prevent the destruction of tight junction proteins by decreasing the number of lipopolysaccharides (LPSs). When LPS binds endothelial cells to toll-like receptors (TLR 2, 4), dendritic cells and macrophage cells are activated, and inflammatory markers are increased. Furthermore, a decrease in gut dysbiosis and intestinal leakage after probiotic therapy may minimize the development of inflammatory biomarkers and blunt unnecessary activation of the immune system. In turn, probiotics improve the differentiation of T-cells against Th2 and development of Th2 cytokines such as IL-4 and IL-10. The present narrative review explores the interactions between gut microflora/probiotics and the immune system starting from the general perspective of a biological plausibility to get to the *in vitro* and *in vivo* demonstrations of a probiotic-based approach up to the possible uses for novel therapeutic strategies.

## Introduction

On 23 February 2004, Time Magazine dedicated the cover to chronic inflammation with a provocative title: *The Secret Killer*. The hypothesis is that, if we think of inflammation from an evolutionary point of view, we are now the victims of our own success. We developed as a species because of our capacity to eliminate dangerous microbial species, but the survival tactics used by our immune system, that was necessary at a time when there were no antibiotics or drainage systems, turned against us. An excess of inflammation and the inability to stop this system can do more harm than good. Chronic inflammation occurs at varying degrees with advanced age in all mammals, regardless of infection and progresses gradually. This is, in part, the cause of many inflammatory chronic diseases (ICDs) and poses a significant threat to human health and longevity. Chronic inflammation follows the failure of the immune system to shut down its reaction to a real or alleged attack. The inability of the body to eliminate an inflammation-inducing agent is the cause of a pro-inflammatory state that can spread all over the body. The health status in our industrialized population is endangered by a plethora of ICD characterized by a widespread and latent low-grade inflammation. These include allergy, certain autoimmune diseases such as Celiac Disease (CeD), Inflammatory Bowel Disease (IBD), Irritable Bowel Syndrome (IBS), obesity and autism spectrum disorders (ASDs), which often tend to share similar environmental risk factors (1) and genetic risk alleles (2).

## Microbial Ecology, Human Evolution, Immune System and Inflammation

People living in today’s urban environments have access to calorie-dense food, minimal physical activity, and high energy balance; moreover, hygiene regimens have decreased, by large, the extent and severity of microbial exposition (3, 4). Certain bacteria, called “old-friends” such as lactobacilli, have been a species of microorganisms that have become part of the human ecosystem for centuries and are usually regarded as harmless to their hosts and in the last decades we are losing our “old-friends”. The immune system evolves and operates in an ecosystem that is an integral part of its natural environment (5, 6) and a dramatic shift of this environment, such as that we are experiencing today, may alter a millennial balance of co-evolution causing a mismatch responsible of an increase of a disease state.

We have evolved in condition with suboptimal nutritional status and significant levels of microbial contact, while today, we are an over-nourished, under-infected industrial population (6–8). Given the crucial significance of new environments in shaping the development and functioning of the human immune system, the make-up of the intestinal microbiota plays a significant role in its training. Emerging literature indicates significant variability in human immune growth and function, and the process of microbial colonization starting at birth and consolidated in the first thousand days is an essential determinant of individual immune responses.

The hygiene hypothesis supports a negative correlation between infancy microbial exposures and inflammation in adults: low rates of microbial contact soon in life, appear to contribute to the dysregulation of immune function and regulatory processes that raise the risk of ICD later in life (9–11). Frequent yet temporary interactions with bacteria can be significant in this process, such as local environment can affect the make-up of resident bacterial populations in the human intestine that have long-lasting effects on immunity (12). Mechanisms behind these adaptations are not simple and include the regulation of T cells and the balance between pro-and anti-inflammatory cytokine composition (10, 13).

Conceptually, microbial exposures play a significant role in developing successful regulatory networks in vulnerable periods of childhood immune shaping. Less hygienic conditions increase the incidence and abundance of bacterial sources, resulting in an increased ability to control inflammation. Differently, extra hygienic conditions reduce the extent and severity of bacterial contact in children, restrict incentives for turning on and off of inflammatory processes during crucial phases of immune shaping. The effect is a pro-inflammatory phenotype. Later in life, when inflammatory processes are activated, there is an inadequate anti-inflammatory regulation resulting in a persistent chronic state of activation.

Early life dietary and bacterial exposures, facilitate the growth of more effective immune defenses and its ability to hone the inflammatory regulation mechanisms encouraging the emergence of an effective adaptive immune system. These findings underscore the function of infant environments in influencing several facets of an immune-phenotype and point to the significance of bacterial exposures.

## Immune System and Gut Bacteria

We lived in a bacteria world and developed a symbiotic state with our bacteria that may raise safety issues. The large bacterial population in the lower intestine are in close contact with human structures, and to separate the inside from the outside, there is just a single cell layer on a vast surface. This close contact with bacteria, if not controlled, can give rise to threatening health complications. Therefore, the immune system has put in place mechanisms to maintain a symbiotic bond between its guests. The need to maintain a homeostatic relationship with the microbiota has been a driving factor force in the evolution of the human immune system and to keep the gut microbiota for its essential role in host metabolism and functions (14).

This alliance was reached by a fine-tuned contact modulation between gut bacteria and intestinal epithelial cells and by limiting the possible entrance of bacteria trough the mucosal layer. In case of an occasional breach in the gut barrier, microorganisms can infiltrate the intestinal epithelial cell and evoke an immune response guided by mucosal dendritic cells (DCs) able to induce a defensive secretory Immunoglobulin A (IgA) response (15).

Studies in germ-free and colonized mice showed how significant is the impact of the gut bacteria on the shaping of the immune system. It has recently become clear that human commensal organisms have an impact on the structure of gut T lymphocyte function. The healthy balance in the intestinal district is preserved by the circuitry of monitoring mechanisms between potentially pro-inflammatory cell [T helper (Th) cells secreting interferon (IFN)-*γ*, Th17 cells that secrete interleukin (IL)-17, and IL-22], and anti-inflammatory Foxp3+ receptor T-cells [Regulatory T (Treg) Cells)]. Many bacteria can stimulate the anti-inflammatory fork of the adaptive immune system by controlling Treg maturation or by driving IL-10 production. In instance, Atarashi K et al. have demonstrated that a mix of 46 strains of Clostridia clusters IV and XIVa, colonizing gnotobiotic mice can induce a local and systemic Treg cell response (16). Besides, *Bacteroides fragilis* polysaccharide A (PSA) elicit an IL-10 response in gut T cells that prevent the spread of TH17 cells responsible for derangement of the intestinal wall (17). On the other hand, mutant *Bacteroides fragilis* without PSA has an opposite inflammatory behavior and does not induce IL-10. It has become clear that the impact of gut bacteria and T cell co-operation goes beyond the intestinal site and can have an effect on systemic in areas far from the gut (18). The type of microbial establishment is the driving force in numerous mouse models of autoimmune conditions such as arthritis and experimental autoimmune encephalomyelitis (EAE) in which the disease state of activation is dependent on gut microbiota composition. Lee YK and Wu HJ et al. in a model of Th17 cell-dependent arthritis and EAE, have demonstrated that colonization with segmented filamentous bacteria is able to set off the disorder (19–21) indicating that the gut bacteria have a systemic immune effect that extends far from the mucosal site.

Under normal conditions, careful regulation restricts excessive inflammation and maintains an immune balance (22–25) that if lost increases the susceptibility for ICDs (26–28). The disruption of nutrition–microbiome–host–metabolism interrelationships is commonly functionally described as “dysbiosis” that is a is a recurring element of various ICDs (23, 25).

## Establishment of the Human Gut Microbiota

The human gut microbiota develops in composition and function in the first years of life (29) reaching a firm microbial population by the second year and an adult-like profile by the time of 4 years (30). This steady-state is driven by a complex interplay between climate, food, microbes, and host factors (31, 32). Babies born by cesarean section (CS) develop a microbiota that resembles that of the skin of the mother. Conversely, the microbiota of a vaginally delivered baby is close to its mother’s vagina and characterized by lactobacilli and bifidobacteria (33). The capacity of cross-talking between microbes and the immune system is mandatory to allow bacterial priming and maturation of the immune system, considering that 70% of immune cells are resident in the gut. In addition to CS (34–36) antibiotics (31), breastfeeding (34, 36), and solid food introduction (29), guide the development of gut microbiota. Infants born with CS are more likely to have respiratory disease (37) and are at increased risk for atopy/asthma (38), obesity (39), and type-1 diabetes (40).

## Western Lifestyle, Diet and Chronic Inflammation

Western lifestyle can trigger an aberrant innate immune activation and disease pathogenesis; recent data suggest that the western lifestyle can set off a systemic inflammatory arrangement leading to health issues typical of industrialized countries.

Among western lifestyle, the main contribution is the adherence to western-type diets (WDs) progressively expanded to low-income nations, with a concurrent increase in ICDs in areas of the planet where these diseases were rare (41, 42). The habitual consumption of WDs can impact on host metabolism and fitness by favoring weight increase, alteration of lipid profile, energy metabolism, and immune activation and promoting several chronic metabolic disorders (obesity, type-2 diabetes mellitus, cardiovascular diseases, and neurodegenerative and autoimmune diseases).

WDs are high in simple sugars, white flour, salt, processed meats, animal fats and food additives, and poor of fiber, minerals, vitamins, or antioxidants (43, 44). The immediate consequence is a rapid weight gain (45). A quick look to the ingredients of WDs allows identifying the components able to elicit an inflammatory response: cholesterol, refined sugars, dairy products, and saturated fatty acids (SFAs) (46). The composition of gut bacteria under the pressure of WD undergoes a profound modification that results in derangement of the eubiotic state. This new microbial balance is responsible for the secretion of microbial metabolites that can reach the systemic circulation, can cause derangement of intestinal permeability that can potentially induce endotoxemia and systemic inflammatory (47). Tanoue T. et al. have demonstrated that mice fed with WD develop a profound dysbiosis leading to immune dysfunction resulting in: a) decrease in mucous secretion, b) loss of secretive IgA function, c) inhibition of Treg cells producing IL-10, d) impaired barrier integrity, e) loss of immune homeostasis, that is the premise for the future onset of ICD (autoimmune and allergy) (48).

Red meat, eggs, and milk-based products coupled with a low intake of fruits and veggies are connected with changes in the intestinal microbial composition, gut inflammation (49). Dietary L-carnitine is metabolized into a metabolic product known as trimethylamine N-oxide (TMAO). The production of this metabolite is dependent on a microbial fermentation occurring in anaerobiosis (49). TMAO is able to activate macrophage and platelet inflammatory response causing endothelial dysfunction, vascular inflammation, and ultimately increasing the risk of cardiovascular disease (50). Caesar et al. demonstrated that WD, particularly saturated fats, influences gut microbiota and causes the inflammation of adipose tissue; conversely, unsaturated fats protect animals from this complication. Interestingly, the authors show that the health-promoting effect is mediated by the microbiota since the transfer of gut microbes from unsaturated to saturated fat-fed mice reduces white adipose tissue inflammation (51).

Is it possible to compensate for the loss of the optimal microbiota with the use of probiotics? The answer is “we still do not know,” but emerging data show that in a not too distant future, selected strains of probiotics may be used to direct the immune response towards the path we need.

## Gut Dysbiosis

The gut microbiota is a microbial ecosystem that has a dramatic role in human health, and it is particularly challenging to define a healthy microbiota; however, this is of great importance if we aim to prevent/correct the alterations of its composition that can impact on our health. There is no “one normal microbiota” since the degree of variability makes it impossible to define what is normal; however, there are some characteristics that can help us to determine a microbiota as healthy: increased diversity, gene richness, amount of butyrate-producing species and resilience.

Resilience is the ability of an ecosystem to withstand alteration under stress or to promptly and thoroughly bounce back from the interference. Therefore, a healthy microbiota is able to recover and go back to baseline after a perturbation (such as an antibiotic treatment) as a result of its resilience avoiding the institution of a new balance and a shift into dysbiosis, with a negative effect on human health (52). As we live, our gut microbiota encounters several perturbators (unhealthy diet, antibiotics, drugs, alcohol, intensive exercise, and pathogenic bacteria). If it is not able to oppose to these attacks, a permanent change occurs leading to a new balance which might not be healthy: this is what we call dysbiosis.

Gut dysbiosis relates to differences in the composition and activity of the gut microbiota that, through qualitative and quantitative shifts in the gut bacteria itself, changes in its metabolic activities and/or changes in its local distribution, have detrimental effects on host health (53). Certain commensal bacteria inhibit the growth of opportunistic pathogens. For instance, during lactose fermentation, *Bifidobacterium* decreases intestinal pH, hence preventing colonization by pathogenic *Escherichia (E.) coli*. The metabolites of commensal bacteria also directly inhibit the pathogens’ virulence genes. If antibiotic treatment subsequently disrupts the resident microbial populations, it may cause inflammation. For example, in colitis mouse model induced by dextran sulfate sodium (DSS), antibiotic increases the abundance of *E. coli*, encouraging the pathogen’s systemic circulation, thus inflammasome activation (54).

Analyzing the importance of dysbiosis-driven diseases in humans, the immune system–gut microbiota crosstalk is of supreme significance. By enhancing the mucosal barrier and fostering innate immune system, commensal bacteria avoid pathogen invasion and infection. It is well-known that the gut microbiota activates the growth and development of the immune response and plays a vital role in immune cell maturation, too. Gut microbial diversity and abundance have been recognized as powerful determinants of host wellbeing, and variations in diversity have been correlated with several human disorders. However, several studies have shown that intestinal microbiota, through complex interactions between intestinal microbiota, host metabolism, and immune systems, directly contribute to the pathophysiology of specific diseases (55). The relationship between gut dysbiosis and inflammation of the mucosa is either a cause or a consequence of dysbiosis, or a combination of both, with one study indicating that intestinal microbiota is necessary for the initiation and progression of inflammation of the mucosa in germ-free mice.

The mucosal barrier formed by intestinal epithelial cells serves as a defense measure, isolating bacteria from host immune cells. Altering the epithelial membrane raises the sensitivity to infection and the delivery of microbial metabolites to the host. Gut dysbiosis not only decreases the stability of the mucosal barrier but also disrupts the immune system, inducing oxidative stress and inflammation. Over time, chronic intestinal dysbiosis and bacterial translocation of bacteria can increase the prevalence of a variety of diseases. Below, we illustrate conditions with detailed research and clinical models relating the mucosal immune system and inflammation, the occurrence of disease and severity.

## Probiotics and Inflammation

Several strains of probiotics have been shown to exert multiple and varied effects on the host and its immune system (56). Their essential role in inflammatory regulation has been well elucidated in several *in vitro* and *ex vivo* models and in germ-free mice showing the failure of the systemic immune regulatory networks, which triggers a cascade of events leading to an inflammatory response.

**Figure 1:**
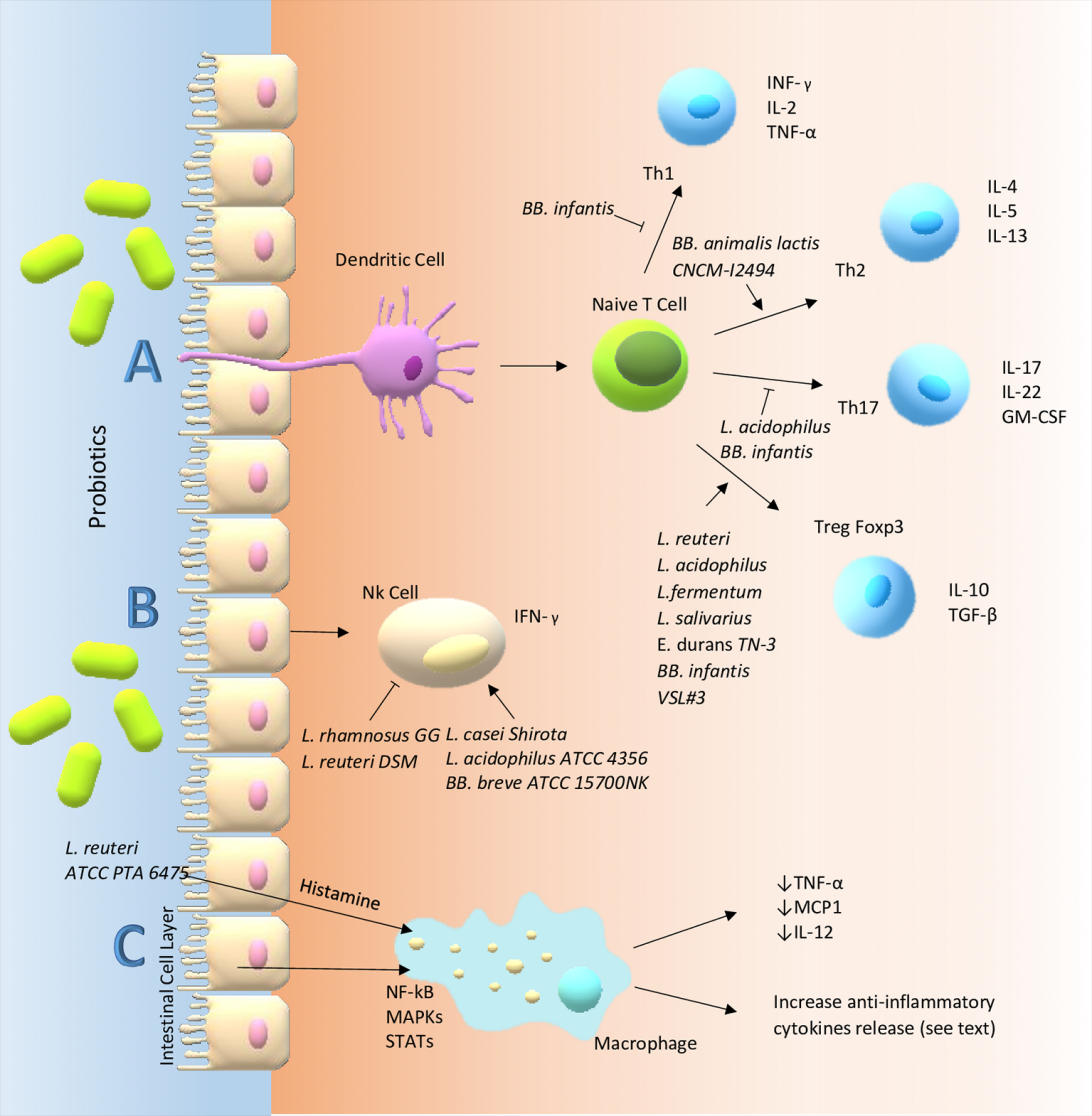
Main probiotic immune-modulatory pathways in the gut. **(A)** T cells are considered the masters of inflammation in that they can differentiate into different pathways promoting or suppressing inflammatory response. However, their fate requires interaction with other cells: for instance, dendritic cells. Probiotics can influence these communications through membrane receptors *e.g.* PRRs. In particular, TLR-6 and TLR-2, a member of PRRs, expressed on sentinel cells such as macrophages and dendritic cells, might be able to decrease the Th17 polarization and skew T-cells toward Treg subpopulation and production of high levels of IL-10 and lower levels of TNF-α, reducing the inflammatory state which could be one of the mechanisms involved in the immune modulatory effect of probiotics in inflammatory intestinal diseases. Probiotics seem to redirect the Th2 response, characteristic of atopic patients, towards a Th1 type through increased secretion of IFN-*γ* and a decrease in the IL-4, IL-13, and IgE production with improvement of allergic predisposition and reactions. In the gut, probiotics may activate B cells in the lamina propria that become IgA-producing plasma cells. The IgA, a major functional component of the humoral adaptive immune system specialized in mucosal protection and a first line of defense against gastrointestinal infections, is transported across the epithelial cells and once it is secreted in the gut lumen it can bind to the mucus layer covering them. **(B)** NK cells are intermediate cells between innate and adapted immunity. They seem to act in different ways through an interplay with intestinal epithelial cells, DCs and T cells. Probiotics can modulate their behavior for instance through the secretion of IFN-*γ*. **(C)** Finally, probiotics exert their functions by altering intracellular pathways of immune cells (e.g. macrophage) through kinases (such as MAP kinase cascade) which in the end activate or suppress transcription factors: STAT, NF-*κ*B, Jun-1, Fos. On the other hand, probiotics may act on the same pathway through the metabolism of histamine acting on H2-receptors of antigen presenting cells and inducing a reduction of pro-inflammatory cytokines like TNF-α, IL-12 and monocyte chemotactic protein-1.

Specific bacterial strains can act on the gut luminal environment, intestinal mucosal barrier, and they can regulate the mucosal immune system. Probiotics may affect different cells involved in the innate and acquired immunity, for instance, DCs, monocytes, Natural Killer (NK) cells, macrophages, lymphocytes and epithelial cells (see **Figure 1**). In particular, they may activate the pattern recognition receptors (PRRs) expressed on immune (*i.e.* M cells in Peyer’s patches) and non-immune cells (*i.e.* intestinal epithelial cells). Among PRRs, TLRs are the most studied which can activate signaling cascades that lead to cell proliferation and cytokine releasing in order to modulate the immune system (57). Moreover, some specific strains secrete substances that may induce the activation of immune cells. In particular, M cells phagocytize or internalize the probiotic and the antigenic components derived from its metabolites, to form endosomes.

Specific probiotic strains can activate DCs, which transport the antigens to local lymph nodes with the following release of IL-10 and IL-12. Here, DCs induce the differentiation of naive T and B cells into their subpopulations, deploying their arsenal of cytokines. In details, naïve Th cells can differentiate into Treg, Th1, and Th2 immune cells, and B cells may shift in plasma cells, playing an active role in humoral responses, or regulatory B (Breg) cells, involved in the production of tumor growth factor (TGF)-*β* or IL-10 (58). Furthermore, DCs may stimulate the activation of NK cells through the production of cytokines such as IL-12 and IL-15. Other probiotic microorganisms, in particular several Lactic Acid Bacteria (LAB), may promote IFN-*γ* production by NK cells *via* DCs (59).

Studies have demonstrated that the immune modulation deriving from probiotic bacteria may be due to the release of the anti-inflammatory cytokine in the gut. Nonetheless, the specific molecular interactions between probiotics and host are not well defined (60–62). The most used probiotics in human belong to *Lactobacillus (L.)*, *Bacillus (B.)*, and *Bifidobacterium (BB.)*, but also the genus *Saccharomyces (S.)* is widely adopted in commercial products. Specific strains of *Lactobacillus* may modulate the cytokine production by immune cells, and *Bifidobacterium* induces tolerance acquisition (63). Such different regulatory activities by each probiotic strain are linked to their structure, to the spectrum of mediators released, and to the various pathways that are simultaneously activated (64, 65).

The anti-inflammatory effects of probiotics have been studied *in vitro*, *ex vivo* and in animal experiments to evaluate cytokine production and immune cell proliferation. In the following sections, we report the recently published evidence of the anti-inflammatory effect of several probiotic strains.

### *In Vitro* and *Ex Vivo* Studies

The interplay mechanisms between probiotics, intestinal cells, and immune system are summarized in **Table 1**; we report the *in vitro* and the *ex vivo* studies published in the last five years. The complex interplay between gut microbiota and immune system, shaped by millions of years of evolution, needs to be deeply explored, in consideration of the rapid technological development, which allows the use of increasingly refined techniques. It is important to underline that these methods are basically used as screening tools. The results of *in vitro* and animal studies are not definitive requiring further confirmation by double blind placebo controlled clinical trials.

**Table 1:** *In vitro* and *ex vivo* studies of different probiotic strains effects in modulating inflammation.

Reference	Probiotic strains	Doses/different concentrations of probiotic suspensions	Studied model	Effect on cytokine patterns and inflammatory mediators
B. K. Thakur et al. (66)	*L. casei* Lbs2	1 × 10^9^ CFU/ml	*In vitro*	↑ IL-10 and TGF-*β*
S. Eslami et al. (67)	*L. crispatus* SJ-3C-US	200 µg/ml	*In vitro*	↑ IL-10 and TGF-*β*
Y. Haileselassie et al. (68)	*L. reuteri* 17938	5% CFS	*In vitro*	↑ FOXp3 and IL-10
M.A Johansson et al. (69)	*L. rhamnosus* GG *L. reuteri* DSM 17938	2.5% CFS	*In vitro*	↓ INF-*γ* Inhibited T cells and NK cell activation
I. M. Smith et al. (70)	*K. marxianus* *S. boulardii*	1 × 10^7^ cells/µl	*In vitro*	Both: ↑ IL-12, IL-1β, IL-6, IL-10 *S. boulardii*: ↑ INF-*γ*
C. Ren (71)	*L. plantarum* CCFM634 *L. plantarum* CCFM734 *L. fermentum* CCFM381 *L. acidophilus* CCFM137 *S. thermophilus* CCFM218	Cells to bacteria ratios of 1:10 1:20 1:40	*In vitro*	Stimulated TLR2/TLR6 heterodimer receptor
D. Compare (72)	*L. casei* DG	1 × 15^7^ CFU	*Ex vivo*	↓ IL-1α, IL-6, IL-8 Increased TLR4 expression
S. De Marco et al. (73)	*L. acidophilus* ATCC 4356 *L. casei* ATCC 334	1 × 108 CFU/ml Cells treated CFS (10%v/v)	*In vitro*	Both: ↓ TNF-α ↑IL-10 *L. casei*: ↓ IL-1β
M. Sichetti et al. (74)	*L. rhamnosus* *BB. lactis* *BB. longum* (Serobioma)	2 × 10^6^ PBMC: 2 × 10^5^ CFU), (2 × 10^6^ PBMC: 2 × 10^6^ CFU), (2 × 10^6^ PBMC: 2 × 10^7^ CFU) *	*In vitro*	↑ IL-10 ↓ IL-1β and IL-6
M. Kawano et al. (18)	*L. helveticus* LH2171	10 µg/ml	*In vitro*	↓ IL-6 and IL-1β Inhibited NF-*κ*B/MAPKs
S.C. Li et al. (75)	*L. acidophilus* and *BB. animalis* subsp. lactis	Cells to bacteria ratios of 1:1, 1:10	*In vitro*	↓ IL-8 Inhibited p-p65 NF-*κ*B, p-p38 MAPK, VCAM-1 and COX-2 Increased TLR2 expression
V. Sagheddu et al. (76)	*L. reuteri* LMG P-2748	1 × 108 CFU/ml	*In vitro*	↑ IL-10

#### Cytokine and Immune Cell Modulation

In this section, we present *in vivo* and *in vitro* studies conducted to highlight the immunomodulatory functions of probiotics and the mode of action.

One of the most known effects of probiotics is that of promoting a shift from Th2 to Th1 cells, to decrease allergic reactions. Human peripheral blood lymphocytes and peripheral blood mononuclear cells (PBMCs), in the presence of LAB, are able to increase the IFN-*γ* production by T and NK cells (77, 78). These results are in agreement with an *in vitro* experiment showing that lactobacilli found in fermented food strongly induced pro-IFN-*γ* monokine IL-12 and IL-18 production by human or murine leukocytes (79, 80). The ability to shift toward a Th2 response might be used in atopy as in other types of Th2 based inflammatory diseases.

Cytokine IL-8 is crucial for the recruitment of immune cells during an inflammatory response. Luerce et al., in a model of colitis-recurrence in CACO-2 cells, demonstrated the ability of *L. lactis* NCDO 2118 to reduce the IL-8 secretion induced by IL-1β (81). In reality, also *BB. animalis* subsp. *lactis* and *L. acidophilus* may decrease IL-8 production and the expressions of pro-inflammatory mediators and increase TLR2 expression *in vitro* model. This anti-inflammatory action is attained through the modulation of TLR2-mediated Nuclear Factor kappa-light-chain-enhancer of activated B cells (NF-*κ*B) and mitogen-activated protein kinase (MAPK) signaling pathways in inflammatory intestinal epithelial cells (75). Furthermore, Ren et al. showed that several strains such as *L. plantarum* CCFM634, *L. plantarum* CCFM734, *L. acidophilus* CCFM137, *Streptococcus thermophilus* CCFM218 and *L. fermentum* CCFM381 enhanced TLR2/TLR6 heterodimer receptor in a strain-specific way; this activity is the initiator of an intracellular signaling network with immune-modulating effects (71). In an *ex vivo* study based on lipopolysaccharide (LPS) stimulation in colonic mucosa from post-infectious IBD, *L. casei* DG and one of its postbiotics suppress pro-inflammatory IL-8, IL-1α, IL-6 and TLR-4 expression levels parallel to an increase of IL-10 (72). The use of probiotics has also been studied in necrotizing enterocolitis (NEC) that is one of the leading causes of death in premature newborns. In particular, *L. rhamnosus* HN001 was studied *ex vivo* on human intestinal cells from ileus of NEC infants; the authors have shown that this probiotic (both alive or UV-killed) interacts with TLR-9 and suppresses NF-*κ*B inflammatory pathway *via* TLR-4 inhibition (82).

Among the other immunological players, Treg cells are the masters of immune modulation and tolerance. *L. reuteri* and *L. casei* exert an anti-inflammatory action, upregulating Treg cell activation and IL-10 levels (probably by DC-SIGN receptor on bone marrow-derived dendritic cells (BMDCs). The overall effect is a significant downregulation of pro-inflammatory cascade with inhibition of bystander T cell proliferation. These strains have been used in *in vivo* studies in inflammatory diseases, including Crohn’s disease (CD) and atopic dermatitis (64). This activity on Treg cells has also been demonstrated for a mix of probiotics (*BB. Bifidium, L. casei, L. reuteri, L. acidophilus*, and *Streptococcus thermophiles)* able to downregulate B and T cell responses with a net production of inhibitory cytokines (83).

Mouse BMDC co-cultured with VSL#3 (a probiotic combination of four *Lactobacillus* strains, three *Bifidobacterium* species, and one *Streptococcus* strain) produced high concentrations in IL-12p70, IL-23, and IL-10 (84). A multistrain mixture of *L. rhamnosus*, *BB. lactis* and *BB. longum* induced an increase of the level of an IL-10 and a reduction of pro-inflammatory IL-1 and IL-6 by 70 and 80% respectively in the human macrophage cell line derived from acute monocytic leukemia patients and macrophages derived from *ex vivo* human PBMC (74). Thakur et al. demonstrated that in BMDCs grown with both live and heat-killed Lactobacilli (*L. casei* Lbs2), TLR-2 receptors were triggered on DCs which led to the differentiation of naive Th cells toward Treg cells and production of IL-10 and TGF-*β* (66). Similar results were obtained when human PBMCs were cultured in the presence of *L. crispatus* SJ-3C-US (which is found in vaginal microbiota from healthy females and seems to be protective against vaginitis and Pelvic Inflammatory Disease) (67, 85).

Attention has also been focused on the primers of immunomodulation, *i.e.* innate immunity.

*L. reuteri* ATCC PTA 6475 can suppress Tumor Necrosis Factor (TNF)-α production induced by LPS through the inhibition of MAPK regulated c-Jun and activator protein-1 pathway (86). A probiotic mixture engagement with regulatory CD11+ DC enhances Treg cells rising the levels of TGF-*β*, IL-10, cyclooxygenase-2 (COX-2) and suppress the production of pro-inflammatory IL-17, IFN-*γ*, and TNF-α (83). *L. reuteri* LMG P-27481 is a new strain discovered and studied by Sagheddu et al. This strain is able to induce a significant secretion of IL-10 when exposed to human immature DCs. If compared to other *L. reuteri* strains, it shows higher anti-inflammatory effect. In *in vitro* co-cultures, *L. reuteri* LMG P-27481 was able to control the growth of *Escherichia (E.) coli*, Salmonella and Rotavirus; however, only this strain was able to hinder the growth *of Clostridium (C.) difficile*. Another excellent property correlated to the genetic background of this probiotic is the ability to metabolize lactose which can be of great importance in case of diarrhea (76). Griet et al., in an *in vitro* assay on murine macrophages (model of acute lung injury) stimulated with LPS, demonstrated that *L. reuteri* CRL1098 decreased the production of a) pro-inflammatory mediator such as COX-2, b) nitric oxide synthase (NOS) and c) pro-inflammatory cytokines (TNF-α and IL-6) (87). Moreover, soluble factors produced by *L. reuteri* CRL1098 were also to inhibit TNF-α production by human PBMC (88)

Specific probiotics strains can negatively or positively stimulate NK cells: *L. rhamnosus* GG and *L. reuteri* DSM 17938 inhibit the activation of T cells and NK cells and the release of IFN-*γ* from *Staphylococcus-aureus*-cultured PBMCs (69). On the contrary, heat-killed *L. casei* Shirota, *L. acidophilus* ATCC 4356, and *BB. breve* ATCC 15700NK increase NK cell activity and enhance their activation (62). The different interplay between probiotic, DCs and NK cells clearly reveals how each strain can differently modulate the immune system and the inflammatory responses; the NK/DC balance is a complex and probiotics may be used to exert beneficial effects (57).

Probiotics were also chosen among other microbiota components such as yeasts. It has been recently demonstrated that *Kluyveromyces (K.) marxianus* and *S. boulardii* stimulate DC production of IL-12, IL- 1β, IL-6, and IL-10. Moreover, *β*-glucan, a polysaccharide derived from their cell wall, could positively interact with DC-receptor Dectin-1 leading to the release of IL-1β, IL-6, and IL-10, but not IL-12. When naïve T cells were cultured with DC in the presence of these probiotics, each strain has its own effect: *K. marxianus* promotes Treg cells and secretion of IL-10, while *S. boulardii* induces a Th1 type response with the production of IFN-*γ* (70). Thomas et al. observed that when *S. boulardii* was grown with bone marrow-derived DCs from CD or ulcerative colitis (UC) patients, there was a reduced concentration of TNF-α and an increase of IL-6 and IL-8 resulting in a negative immune modulation and increased levels of TGF-*β* which could help in epithelial barrier restoration (89). The challenge of *S. boulardii* on DCs derived from PBMCs was followed by a reduction of TNF-α and IL-6 and an increase of IL-10, thus blocking T cell activation and promoting the polarization of naive T cells towards Treg cells (90).

#### Anti-Inflammatory Effect of Probiotics Metabolites (Postbiotics)

Specific molecules produced by probiotics can contribute to the improvement of host health by promoting specific physiological functions, in the same manner of live probiotics, although the precise mechanisms are not completely elucidated.

Postbiotic supernatant collected from probiotic bacterial cultures could be used to achieve an immune modulation without the possible risks related to living microorganisms such as infections in immune-deficient patients. It is well known that the effect of probiotics can be mediated by their metabolites, such as short-chain fatty acid (SCFA) in particular propionate, acetate, and butyrate that may exercise anti-inflammatory effects. SCFAs are produced by bifidobacilli, lactobacilli, and several commensal bacteria. These postbiotics exert their action by binding to specific receptors on intestinal epithelial cells. In this way, the NF-*κ*B pathway, Treg cell suppression, and pro-inflammatory cytokine production by neutrophils and macrophages are inhibited; consequently, the inflammation state is prevented, and an anti-inflammatory effect is produced (91, 92). Butyrate may exert a beneficial effect in controlling gut inflammation through the induction of Treg cell differentiation (93). For instance, *L. acidophilus* CRL 1014, recently studied in the Simulator of Human Microbial Ecosystem reactor, has shown to produce SCFAs (94) while *BB. longum* SP 07/03 and *BB. bifidum* MF 20/5 can produce only propionate and acetate (95).

Additionally, an anti-inflammatory effect may be obtained by the interaction with tryptophan (deriving from diet) and indolic acid derivatives (produced by probiotics or intestinal bacteria) with specific receptors expressed on immune cells. All of these molecules have a role in gut homeostasis; in particular, indole-3-propionic acid (IPA) significantly promotes IL-10 production with anti-inflammatory activity and decreases TNF-α release. Both *in vitro* and *in vivo* murine studies have shown how *C. sporogenes* can convert tryptophan into IPA, resulting in a protective effect against dextran sulfate sodium-induced acute UC (96).

In other studies, the whole supernatants from probiotic cultures were collected, looking for the sets of regulatory molecules that might induce in immune cells. *L. reuteri* 17938 is widely used as an adjuvant in infection-associated diarrhea, NEC, and IBD. *L. reuteri* derived supernatant mixed with PBMCs induced CCR7 on DC membranes as well as the production of FOXp3 and IL-10 in Treg cells (68). De Marco et al. found that *L. acidophilus* and *L. casei* supernatant may reduce the TNF-α release and stimulate IL-10 secretion. Furthermore, *L. casei* supernatants may inhibit LPS-induced IL-1β activation, which could explain its positive action in IBD (73).

#### Animal Studies

A plethora of studies on the role of probiotics on inflammation has been performed in animals and most of these in colitis-induced murine models.

Based on the *in vitro* studies, *L. reuteri* LMG P-27481 was orally administered in mice with *C. difficile* induced colitis. This strain obtained brilliant results in reducing *C. difficile* colonization and toxin load; it was able to induce an anti-inflammatory response and the restoration of mucosal barrier function resulting in a general improvement of the histologic lesions. The authors speculate that the distinctive features of this strain might be due to the production of bioactive molecules such as exopolysaccharide and peptidoglycan hydrolases. *L. reuteri* LMG P-27481 might be useful as an adjuvant in *C. difficile* infection and other inflammatory diseases; however, further studies are needed to support its use in clinical practice (76). Park et al. showed that, in a dextran sulfate sodium-induced colitis, mice fed with *L. acidophilus* showed an increase in Treg cells and splenic IL-10 coupled with a reduction of splenic IL-17 and colonic IL-6, TNF-*β*, IL-1β, and IL-17 (97). In a recent study, the administration of *L. fermentum* CQPC04 significantly inhibited pro-inflammatory cytokines production (IFN-*γ*, IL-1β, TNF-α, IL-6, and IL-12), and promoted the release of IL-10 in serum ameliorating the colonic damage (98). Choi et al. demonstrated that the oral administration of *L. plantarum* strain CAU1055 significantly decreased the levels of inducible NOS, COX-2, TNF-α, and IL-6 (99). At the intracellular level, decreased phosphorylation of STAT3, leading to suppression of IL-17 and TNF-α and, consequently, of IL-23/Th17 axis, was demonstrated following administration of *L. acidophilus* to mice with UC (100). In a study on the trinitrobenzene (TNBS) model of colitis, *L. reuteri* ATCC PTA6475 was able to reduce inflammation through the histamine H2-receptor signaling (101). Besides, in research on colitis mice, Qiu et al. (102), Rodríguez-Nogales et al. (103), and Kanda et al. (104) showed that probiotics enhanced Th0 cell differentiation to Treg cell and up-regulated IL-10 secretion. *L. rhamnosus* RC007 was studied both in healthy and in TNBS-induced-colitis mice. In the first there was an improvement in the phagocytic activity of peritoneal macrophages. At the same time, in the latter there was a reduction of body weight loss and an improvement of macroscopic and microscopic colonic injury. In both cases, an increase in IL-10/TNFα ratio in the intestinal fluids was found (105). Thakur at al. found that *L. casei* Lbs2 was able to stimulate Treg cells in an experimental mouse model of colitis with an improvement of the severity of the disease (66). A specific strain of *L. plantarum* C88 seems to exert a protective mechanism on liver injury in mice: it down-regulates the levels of IL-8, IL-1β, IL-6, IFN-*γ* and TNF-α and inhibits the NF-*κ*B signaling pathways reducing TLR2 and TLR4 expression (106).

Yang et al. studied the effect of a probiotic mixture (*BB. breve* DM8310, *Streptococcus thermophiles* DM8309, *L. casei* DM8121, and *L. acidophilus* DM8302) on the 5-fluorouracil (5-FU) induced enteropathy. The authors report a positive effect of this mixture and speculate that the possible mechanism could be related to the alteration of the TLR2/TLR4 signaling pathways and the restoration of gut homeostasis. This study shed light on the potential mechanism behind their action in chemotherapy-induced intestinal mucositis (107).

In mice with colorectal cancer induced by dimethylhydrazine, treatment with oral *BB. infantis* suppresses CD4+IL-17+ cells and the secretion of IFN-*γ*, IL-12, and IL-2 from Th17 and Th1 cells, improving mucositis induced by chemotherapy (108).

Good et al. studied *L. rhamnosus* HN001 in NEC-induced mice showing the following positive results: a) when administered this probiotic was not responsible for sepsis (a concern in premature newborns); b) its use, alive or UV-killed, resulted in an improvement in the gross aspect of the gut, lower histology score and attenuation of mucosal cytokine levels with the production of inducible NOS (82). In a different murine model of colitis, the administration of *L. reuteri* 100-23 associated with a diet enriched in tryptophan was able to repolarize gut intraepithelial CD4+ T cells- into Treg cells enhancing immunotolerance (109).

A summary of the mechanisms of probiotics on in-animal models is displayed in **Table 2**.

**Table 2:** Animal studies of different probiotic strains effects in modulating inflammation.

Reference	Probiotic strain	Doses	Studied model	Effect on cytokine patterns and inflammatory mediators
L. Chen et al. (100)	*L. acidophilus*	1 × 10^4^, 1 × 10^5^, 1 × 10^6^, 1 × 10^7^ 1 × 10^8^ CFU/10 g body weight/day	DSS-induced colitis in mice	↓ IL-17, IL-23, TGF*β*1 and TNF-α
C. Gao et al. (101)	*L. reuteri ATCC PTA6495*	5 × 10^9^ CFU/day	TNBS-induced colitis in mice	↓ IL-1β, IL-6 Activation histamine H2-receptor
T. Kanda et al. (104)	*E*. *durans* TN-3	10 mg/day	DSS-induced colitis in mice	↑ Treg and IL-10 ↓ IL-1β, IL-6, IL-17A and IFN-*γ*
C. dogi et al. (105)	*L. rhamnosus RC007*	1 × 10^7^ cells/day	TNBS- induced colitis in mice	↑ IL-10/TNF-*α* ratio
B. K. Thakur et al. (66)	*L. casei Lbs2*	1 × 10^9^ CFU/day	TNBS-colitis-induced in mice	↑ Treg
A. Rodríguez-Nogales et al. (103)	*L. fermentum CECT5716* *L. salivarius CECT5713*	5 × 10^8^ CFU/day	DSS-induced colitis in mice	↓ IL-1β, IL-12 and TGF-*β* ↓ NOS
H. Mi et al. (108)	*BB. Infantis*	1 × 10^9^ CFU/day	colorectal cancer induced by dimethylhydrazine in mice	↓ IL-2, IL-12, and IFN-*γ*
Y. Tang et al. (107)	*B. breve* DM8310 *L. acidophilus* DM8302 *L. casei* DM8121 *S. thermophilus* DM8309	1 × 10^8^ 1 × 10^9^ CFU/Kg/day	5-FU-induced mucositis in mice	↓ IL-6 and TNF-α
J. S. ParK et al. (97)	*L. acidophilus*	8 × 10^8^ CFU/Kg/day	DSS-induced colitis in mice	↑ Treg and IL-10 ↓ IL-17
		CFU/10 g body		
X. Zhou et al. (98)	*L. fermentum* CQPC04	1.5 × 10^9^ 5 × 10^10^ CFU/Kg/day	DSS-induced colitis in mice	↑ IL-10 ↓ TNF-α, IFN-*γ*, IL-1β, IL-6, and IL-12 Inhibited NF-*κ*Bp65, COX-2
S. H. Choi et al. (99)	*L. plantarum* CAU1055	8 × 10^9^ CFU/day	DSS-induced colitis in mice	↓ TNF-α and IL-6 ↓ NOS and COX-2
L. Huang et al. (106)	*L. plantarum* C88	4 × 10^10^ CFU/Kg/day	aflatoxin B_1_-induced liver injury in mice	↓ IL-1β, IL-6, IL-8, TNF-α and IFN-*γ* Inhibited NF-*κ*B
M. Liu et al. (2019)	*L. lactis* ML2018	1 × 10^8^ CFU/day	DSS-induced colitis in mice	↓ NF-*κ*B and MAPK
V. Sagheddu et al. (76)	*L. reuteri* LMG P-2748	1 × 10*^9^* CFU/day	*C. difficile* induced colitis in mice	↑ IL-10

### Examples of Anti-Inflammatory Effects of Probiotics *In Vivo*: From Celiac Disease to Parkinson’s Disease

The anti-inflammatory effect of different strains of probiotics, shown in *in vitro* and animal studies, must pass the test of *“the real-life condition”* to assess their potential use in clinical trials. In the following section, we exploit the potential favorable effects of specific probiotic strains as adjuvants in the treatments of ICD, such as CeD, IBD, IBS, obesity, autism, and Parkinson’s Disease (PD) among others.

#### Celiac Disease

CeD is an autoimmune enteropathy that occurs in genetically predisposed individuals after gluten ingestion (110). Although gluten is the only well-established trigger in CeD, a dysbiosis mainly characterized by a rise of *Bacteroides* spp. and a decrease of *Bifidobacterium* spp (111). has been associated with CeD in several studies. The intestinal microbiota in this condition has been hypothesized to have a role in CeD onset, and an international research (the “Celiac Disease Genetic, Environmental, Microbiome, and Metabolomic Analysis”) is underway to examine its possible contribution. This study is a prospective, longitudinal observational cohort study of newborns born in a family in which at least one member is affected by CeD, planned to elucidate whether microbiota composition, time of gluten assumption, and genetic asset are implicated in CeD pathogenesis (112).

The only proven effective treatment for CeD is long-life Gluten-Free Diet (GFD). However, despite strict dietetic adherence, patients often continue to experience gastrointestinal symptoms. The effect of probiotics has been studied as an adjuvant treatment due to its ability to hydrolyze gluten peptides thus reducing their immunogenicity (113) restore gut microbiota, modulate the immune response, and/or reducing the low-grade inflammation, which often does not completely subside after GFD (114). The effects of probiotics in the modulation of immunity (innate and adaptive), and reduction of gliadin-induced inflammation in CeD are mainly demonstrated by animal models (115–119) and, recently, also in several human studies.

In a recent research, it has been demonstrated that treatment with *L. plantarum* HEAL9 and *L. paracasei* 8700:2 (10^10^ CFU/day for six months) can suppress CeD autoimmunity prior to diagnosis and GFD. In this study, the authors showed a different T cell modulation between the control group and the probiotic group, associated with a lower titers of anti-tissue transglutaminase-IgA in the probiotic group (p = 0.013) (120). Klemenak et al. randomized 49 CeD pediatric patients on GFD, to receive either *BB. breve* strains BR03 and B632 (2 × 10^9^ CFU/day) or placebo and demonstrated that three months administration of probiotic leads to lower levels of TNF-α; however, TNF-α levels increased again three months after completion of the intervention (121). The same effect on TNF-α level has been confirmed by Primec et al. who studied the impact of a combination of two *BB. breve* strains for three months (daily dose: 2 g of DSM 16604 and DSM 24706 in a 1:1 ratio) in 40 CeD patients. Besides the positive effect of TNF-α, the probiotic combination was followed by a restoration of the Firmicutes/Bacteroidetes ratio (122). Similarly, *BB. longum* CECT 7347 (10^9^ CFU for ninety days along with GFD) has a positive effect on TNF-α levels in CeD patients. This strain determined a reduction of activated T lymphocytes, a decrease of *Bacteroides fragilis* and secretive IgA content in stools as compared to placebo (123). The same group studied the efficacy of this strain in rats weaned with gliadins. The authors showed that the treatment with *BB. longum* CECT 7347 (6.0× 10^7^–8.2 × 10^8^ CFU/day) partially counteracted the gliadin-induced changes and improved the inflammation as compared with animals fed gliadin alone. In the same study, the effect was less marked when the animals were sensitized with IFN-*γ*, probably because of worst gut damage (124).

Our group recently conducted a clinical trial in 109 CeD patients strictly adherent to the diet with IBS-like symptoms randomizing to receive either a mixture of five strains of lactobacilli and bifidobacteria [*L. casei* LMG 101/37 P-17504 (5 × 10^9^ CFU/sachet), *L. plantarum CECT 4528* (5 × 10^9^ CFU/sachet), *BB. animalis* subsp. lactis Bi1 LMG P-17502 (10 × 10^9^ CFU/sachet), *BB. breve* Bbr8 LMG P-17501 (10 × 10^9^ CFU/sachet), *BB. breve* Bl10 LMG P-17500 (10 × 10^9^ CFU/sachet)] or placebo for six weeks. We were able to demonstrate that this probiotic combination was effective to reduce the severity of IBS symptoms and to modulate microbiota with increased bifidobacteria (125). The ability of the same probiotic combination to hydrolyze gluten has been recently tested in a model of simulated gastrointestinal gliadin digestion. The authors, using CACO-2 cells, showed that physiological-digested gliadin could be further hydrolyzed into lower molecular weight peptides by probiotic bacteria. As compared to physiological-digested gliadin, able to induce the synthesis, up-regulation and dissemination of zonulin and occludin by IL-6, the probiotic digested peptides did not show this negative activity (126).

At present GFD is the only proven therapy for CeD and probiotics, with their ability to modulate intestinal permeability and decrease inflammatory responses, are a safe and promising additional treatment in CeD. In contrast, the possibility of increasing gluten threshold by the concomitant probiotic use, even if attractive, is not yet applicable.

#### Inflammatory Bowel Diseases

A theoretical basis for using microbiota driven strategies in IBD was the recognition that a misdirected immune system has a fundamental role in IBD; gut microbiota with its specific genetic makeup and environmental factors have significant contributions in IBD pathogenesis (127, 128). Several findings, both on human and animal models, demonstrated the importance of the microbiota–host interaction in both CD and UC. Indeed most IBD patients compared with healthy subjects present a deep dysbiosis with lower diversity, a decrease of anti-inflammatory taxa, increased Proteobacteria (such as *E. coli* and *Klebsiella*), *Ruminococcus gnavus*, Pasteurellaceae, Fusobacteria, *Candida tropicalis* and Veillonellaceae and reduced amount of Firmicutes (especially the potentially protective *Faecalibacterium prausnitzii)*, *Bifidobacterium*, Ruminococci, and Clostridium (129–131). The increased gut permeability, typical of IBD patients, facilitates translocation of different bacteria through the intestinal layer (132). The interplay between microbiota and receptors on epithelial cells leads to a chronic inflammation responsible for worsening of the disease (133).

UC is a colonic inflammatory disease with an incidence of 10–20 per 100,000 people (134) characterized by a diffuse inflammation, limited to the colon which starts at the rectum and spreads proximally. UC pathogenesis is multifactorial and includes a strong genetic predisposition, mucosal immunity and epithelial barrier dysregulation as well as dysbiosis (135).

A recent Cochrane meta-analysis, including 14 studies (865 randomized participants; 12 adults and two pediatric studies) explored the role of probiotics in inducing remission in people with UC. A single strain probiotic was tested in half of the included studies; among those, we found *Escherichia coli* strain Nissle 1917 (EcN) (136), *L. reuteri* ATCC 55730 (137) and *BB*. 536 (138). The other seven trials tested multiple strain combinations. The authors concluded that: 1) probiotics might improve the induction of clinical remission if compared to placebo; 2) there is no difference in remission inducing between probiotics and 5-aminosalicylic acid (5-ASA) (139). In the maintenance of UC remission *L. rhamnosus* GG (140), EcN (141, 142), the yeast strain *S. boulardii* (143) and VSL#3 (144, 145) are the most studied strains compared to 5-ASA. Souza et al. evaluated the role of EcN in mice with colitis: their results showed downregulation of inflammation, lower levels of neutrophils, eosinophils, chemokines, and cytokines. The authors demonstrated an increase of Treg in Peyer’s patches associated with an improvement in clinical and histological disease scores. In the same study, germ-free mice transplanted with feces from mice treated with EcN were protected from DSS-induced colitis (146). A large trial performed by Kruis et al. in 2004 evaluated the efficacy of EcN administration in 327 adults with UC in remission. One hundred sixty-two patients received the probiotic (200 mg once daily), while 165 were treated with Mesalazine (1,500 mg per day for 12 months). No difference was found in the relapse of patients treated with EcN as compared to those treated with mesalazine (33.9 *vs.* 36.4%) (147). The results of a recently published Cochrane metanalysis are less encouraging. The authors included 12 studies (1,473 randomized participants, mostly adults) aimed to compare probiotics *vs.* placebo, probiotics *vs.* 5-ASA and a probiotics mixture plus 5-ASA *vs.* 5-ASA alone. Seven studies focused on a single bacterial strain and five studies on multiple strains. The conclusion was that at present, it is not possible to state if probiotics are able to maintain clinical remission in any of the associations studied (148).

Pouchitis is defined as inflammation within the ileal reservoir and is present in up to 60% of UC patients who undergo an ileal-pouch anal anastomosis. One of the most relevant effects of probiotics in IBD has been obtained in patients with this condition. Forty patients were randomized to receive either 6 g/day of VSL#3 or placebo immediately after an ileal-pouch anal anastomosis and were then followed-up for one year: only 10% of VSL#3 patients *vs* 40% of placebo developed acute pouchitis. The conclusion was that VSL#3 is effective in pouchitis prevention (149). Moreover, two RCTs have demonstrated that VSL#3 is also useful in patients with acute pouchitis for maintaining remission. Patients on VSL#3 had no relapse as compared to 6% in patients treated with placebo (150, 151). Git VSL#3 treatment led to a decrease in pro-inflammatory cytokines, increase in Treg cells (152), reduced permeability, and gut microbiota modulation (153).

CD is a chronic inflammatory disease that can occur in any segment of the gastrointestinal tract characterized by massive inflammation that is typically segmental, asymmetrical, and transmural. The possible beneficial effect of probiotics arises from the different possible mechanisms of action: 1) balance in the composition of the gut microbiota, 2) inhibition of enteric pathogens, 3) degradation of bacterial antigens, 4) blockade of inflammatory mediators, and 5) stimulation of local immunity. Compared to UC, in CD gut microbiota is unstable with a decrease of butyrate-producing bacteria species such as Faecalibacterium, Methanobrevibacter, Christensenellaceae, and Oscillospira. Manichanh et al. demonstrated, in CD patients, an increase in Bacteroidetes and Firmicutes ratio compared to the general population (154). The composition of gut microbiota seems to be influenced by the inflammation site: patients with colitis presented an increase in Firmicutes, while those with ileitis presented a lower diversity (155). One-third of CD patients presented an increased number of mucosa-associated adherent-invasive *E. coli*; these strains can cross the mucosal layer causing high amounts of TNF-α production (156, 157). Several meta-analyses report few or no effect of probiotics with exception limited to the prevention/treatment of paucities (158, 159).

Because of the limited-certainty evidence it is not possible to draw firm conclusions on the potential effect of any specific probiotic strain (or combinations) in reducing clinical relapse or inducing remission in IBD patients. The lack of evidence of probiotic efficacy in IBD could be related to the failure in identifying the ideal strain (or combinations), to possible protocol of treatment bias or because the intervention is started too late in the course of the disease when the ‘pathogenic’ microbiota is already established.

#### Irritable Bowel Syndrome

IBS is one of the most frequent functional gastrointestinal disorders, with an estimated prevalence of approximately 11% in adults and from 1 to 5% in children. Recurrent abdominal pain, changes in bowel habits, abdominal distention (160, 161) are the most frequent symptoms of IBS. The etiology is still poorly understood but seems to be multifactorial and includes altered intestinal motility, visceral hypersensitivity, abnormal gut–brain interaction, dysbiosis, and low-grade inflammation. In IBS, gastrointestinal dysbiosis has been linked to an increased mucosal permeability interfering with intestinal homeostasis and thereby increasing low-grade gut inflammation and stimulating cellular and mucosal immune activation (162). It has also been speculated that microbiota alteration can affect gut motility and lead to enteric nervous system dysregulation (163). Even though gut microbiota alterations seem to be crucial in IBS, no uniform gut microbiota pattern has yet been demonstrated. The existing inconsistencies among currently available data may be attributed to several factors, including heterogeneity of gut microbiota study methods and individual microbiota variability.

Recently, Tap J et al. have proposed a microbiota signature related to IBS severity in adults, characterized by low microbial richness, lack of Methanobacteriales and Prevotella and increase Bacteroides (164). A meta-analysis performed on 13 studies involving 360 IBS patients and 268 healthy controls demonstrated a decrease in the abundances of *Lactobacillus*, *Bifidobacterium*, and *Faecalibacterium prausnitzii* (165). In a recent systematic review of 22 articles, Lactobacillaceae, *Enterobacteriaceae*, and *Bacteroides* were more abundant while, uncultivated *Faecalibacterium*, *Clostridiales*, and *Bifidobacterium* were reduced in IBS patients (166).

Studies performed on intestinal biopsies of subjects with post-infective IBS have demonstrated an increase in lymphocytes, mast cells, and inflammatory cytokine possibly related to a dysbiotic microbiota (167, 168). Recently Kim et al. studied the effect of a probiotic multistrain mixture (5 × 10^9^ viable cells *Bifidobacterium longum* BORI, *Bifidobacterium bifidum* BGN4, *Bifidobacterium lactis* AD011, *Bifidobacterium infantis* IBS007, and *Lactobacillus acidophilus* AD031 three times per day for eight weeks) in patients with diarrhea-predominant IBS, using a metabolomic approach. The authors were able to demonstrate that this probiotic mixture improves IBS symptoms and modifies the levels of urinary metabolites related to gut inflammation (169). O’Mahony et al. performed a clinical trial in 77 IBS patients reporting an abnormal IL-10/IL-12 ratio at baseline, suggestive of a pro-inflammatory state. The patients were randomized to receive, for eight weeks, a malted milk drink added either with *L. salivarius* UCC4331 or with *BB. infantis* 35624 (10^10^ live bacterial cells each) or with placebo. Only the treatment with *BB. infantis* 35624 resulted in symptom improvement and normalization of the IL-10/IL-12 ratio (170). Finally, a multispecies probiotic combination (25 billion active bacteria *L rhamnosus* LR5 3 × 10^9^ CFU; *L. casei* LC5 2 × 10^9^ CFU; *L. paracasei* LPC5 1 × 10^9^ CFU; *L. plantarum* LP3 1 × 10^9^ CFU; *L. acidophilus* LA1 5 × 10^9^ CFU; *BB. bifidum* BF3 4 × 10^9^ CFU; *BB. longum* BG7 1 × 10^9^ CFU; *BB. breve* BR3 2 × 10^9^ CFU; *BB. infantis* BT1 1 × 10^9^ CFU; *S. thermophilus* ST3 2 × 10^9^ CFU; *L. bulgaricus* LG1; and *Lactococcus lactis* SL6 3 × 10^9^ CFU) was used to test the possibility to reduce inflammation in IBS patients. One-hundred-seven patients were randomized to the probiotic mixtures (two times a day) or placebo: after eight weeks of treatment, no difference was found in IBS symptoms score, in fecal calprotectin level and high sensitivity C reactive protein between the two groups (171).

Several meta-analyses demonstrate the positive effect of probiotics (single strains and multistrain) in the management of IBS (172, 173) although a single strain or combination has not been definitively identified. The beneficial effect is related to the immune-modulating effect of probiotics on the regulation of anti-inflammatory/pro-inflammatory cytokines that cannot be exclusive of a particular species (170, 174–176).

We recently reviewed the guidelines published by several scientific societies on probiotics in IBS and concluded that in adults, taken as a group, probiotics could ameliorate global symptoms; however, at present no recommendations regarding individual species, preparations, or strains can be made because of limited and conflicting data. In pediatric IBS, current evidence shows efficacy of *L. rhamnosus* GG, *L. reuteri* 17938 and VSL#3, thus supporting their use (177).

#### Obesity

Obesity is defined as excessive/abnormal fat accumulation with adverse health consequences. Obesity and its metabolic complications represent a relevant health problem all over the world. The prevalence of obesity is three times higher since 1975, and more than 13% of the world’s population is obese at present (178). The pathogenesis of obesity is multifactorial and includes hormonal, genetic, and environmental factors. However, growing evidence shows that microbiome influences the energy balance contributing to obesity pathogenesis and its associated complications (179).

Gut microbiota is involved in energy homeostasis by extracting energy from foods through fermentation processes. It has been speculated that the increased energy extraction (especially for plant-derived complex carbohydrates) could have been an advantage in conditions of limited food availability. However, nowadays the increased availability of food and the changes in the proportion of diet components (increased intake of fat and sugar and reduction of plant-derived carbohydrates) can be responsible for a negative effect of our microbiota on human health (180).

It has been reported that the Firmicutes to Bacteroidetes ratio is altered in obese people, and this seems to promote the energy extraction from foods and storage (181). Changes in the intestinal microbiota in response to weight-reducing diets have also been documented (182). On the contrary, gut microbiota alterations can be associated with increased obesity risk (183). A meta-analysis showed that a reduced count of *Bifidobacterium* during early infancy is more often found in obese children as compared to normal weight controls (184).

A developing term in the field of innate immunology is that of metabolic endotoxemia. Metabolic endotoxemia is described as a subclinical increase in circulating endotoxin levels that although not noticeable in clinical settings, plays a significant role in the etiology for many chronic diseases. Several studies have shown an alteration of the intestinal permeability that may potentially trigger the metabolic endotoxemia. Once in the circulation LPS bind to LPS-binding protein promoting the activation of inflammatory pathways, including NF-*κ*B and subsequent cytokine release such as IL-6 and TNF-α, that in turn lead insulin resistance in several tissues (185). It has been hypothesized that targeted microbiota interventions might be used for the prevention and treatment of obesity and associated metabolic conditions (186). The demonstrated effect of probiotics in the regulation of intestinal permeability is an attractive option and worth considering further, especially at the light of data from animal studies where probiotics have shown the ability to improve intestinal permeability and metabolic and inflammatory status (187, 188).

Different authors have shown that some strains of *Bifidobacterium* and *Lactobacillus* can prevent obesity across several studies in animals and human, including *L. rhamnosus, L. casei, L. plantarum, L. gasseri, BB. infantis, BB. longum, and BB. breve* (189). The administration of *L. plantarum* TN8 can induce an increase of anti-inflammatory IL-10 levels as well as a decrease in pro-inflammatory IL-12, IFN-*γ*, and TNF-α in diet-induced obese mice (190). Miyoshi et al. performed a study in mice evaluating the effect of *L. gasseri* SBT2055; they showed that *L. gasseri* significantly influenced fat accumulation, reduced weight gain, and modulated adipose tissue pro-inflammatory cytokines (191).

Park et al. performed a study in diet-induced obese mice on the effect *L. curvatus* HY7601 *and L. plantarum* KY1032 (5 × 10^9^ CFU/day for more than two months): probiotics decreased fat accumulation and reduced BMI. Moreover, the authors observed that, in mice receiving probiotic treatment, pro-inflammatory genes in the adipose tissue (IL1β, TNF-α, IL6, and monocyte chemotactic protein-1) were downregulated. In contrast, fatty acid oxidation-related genes were upregulated in the liver (192). One month administration of *S. boulardii* in obese and type 2 diabetic mice has been shown to reduce obesity, hepatic steatosis, fat mass, and inflammation with a concomitant effect on gut microbiota composition (increased in Bacteroidetes and a decreased in Firmicutes, Proteobacteria, and Tenericutes) (193).

Bernini et al. showed that *BB. lactis* HN019 (80 ml of the probiotic milk containing on average 3.4 × 10^8^ CFU/ml) in patients with metabolic syndrome led to reduced weight gain and modulation of cytokines such as IL-6 and TNF-α (194). The supplementation with *L. reuteri* V3401 (5 × 10^9^ CFU for three months) in patients with metabolic syndrome resulted in IL-6 and soluble vascular cell adhesion molecule 1 (sVCAM-1) decrease associated with a rise in the proportion of Verrucomicrobia (195).

Several systematic reviews and meta-analyses evaluating the role of probiotics in obesity have been published, and most of them have shown a BMI decrease in enrolled patients who were supplemented with several probiotics strains (196, 197); however, other trials failed to demonstrate this effect. Recently the effects of prophylactic *BB. lactis* BS01 and *L. acidophilus* LA02 supplementation (2 × 10^9^ CFU for six weeks) were studied. On anthropometric measures in healthy, young females: no significant effects were found on all anthropometric measurements (198).

Given the current epidemic of obesity plaguing Western society, a call is necessary for feasible, available, and safe treatments to prevent and fight against it. Even though obesity pathogenesis is multifactorial and highly complicated, recent literature suggests microbiota alterations as the primary contributor to its development and associated metabolic and inflammatory abnormalities. Consequently, gut microbiome modulation to preserve a stable, consistent metabolic environment may be helpful in preventing and as additional treatment in obese people.

#### Autism

ASDs are a variety of developmental disabilities that usually appear in the first few years of life and manifest in several ways with different grades of severity from mild impairment to complete inability to live an adequate social and personal life. The incidence of ASD is increasing, although it is still unknown if this is secondary to an increase in social awareness and earlier diagnosis or to a real rise in prevalence secondary to modified environmental conditions (199). The etiology is still unknown: genetic, epigenetic, environmental, and infectious factors have been identified as possible cofactors that enter in the pathogenesis of this multifactorial disorder. It is well established that there is a strong connection between the brain and gut: the so-called brain–gut axis. This connection implies that these two organs, although so different, influence each other’s development and function. In particular intestinal microbiota is able to send signals to the central nervous system *via* intestinal epithelial, bowel neuronal and immune cells. Germ-free mice develop different alterations of neurotransmitter turnover, neuroinflammation, neurogenesis, and neuronal morphology (200). In ASD patients, the intestinal microbiota is altered: as compared to healthy peers higher concentrations of pathogenic Clostridium bacteria (201), a decreased *Bacteroides*/Firmicutes ratio, and increased *Lactobacillus* and *Desulfovibrio* species (202, 203) have been shown. This altered microbiota creates an inflammatory environment with the release of cytokines leading to a disruption of the mucosal barrier functions.

It has been reported that alterations in cytokine levels and immune dysregulation are frequent in ASD patients. IL-6, macrophage chemoattractant protein-1 and TNF-α play a role in cerebral inflammation (204) and a higher concentrations of these chemokines along with IFN-*γ*, IL-1β, are present in ASD patients (205). In a murine model of ASD, de Theije C et al. investigated the relationship between gut microbiota and autism-like behavior in mice *in utero* exposed to valproic acid. As expected, the authors demonstrated a change in the Firmicutes and Bacteroidetes phyla in the offspring supporting that an intestinal phenotype is associated with autism-like behavior with preponderance in male offspring and associated with boosted levels of cecal butyrate and ileal neutrophil infiltration and inversely correlated with serotonin gut levels (206).

In a recent study performed in an animal model of obsessive-compulsive disorder, the effects of *L. casei* Shirota consumption has been studied. Behavioral tests demonstrated the reduction of obsessive-compulsive disorder symptoms in mice after *L. casei* Shirota treatment, and the authors demonstrated that this is secondary to the regulation of serotonin-related genes expression (207). In a randomized placebo-controlled double-blind study, ASD children were treated with *L. plantarum* WCSF1 for three months. An increase in enterococci and lactobacilli and a reduction in Clostridium cluster XIVa were found. This resulted not only in an improvement of the intestinal symptoms but, more importantly, in an increase in behavioral scores (208). By treating ASD children, their siblings and neurotypical children with a mixture of probiotics (*Lactobacillus*, *Bifidobacterium* and Streptococcus strains) for four months, Tomova et al. found a decrease in TNF-α levels, a primary inflammatory cytokine, reduced *Bifidobacterium* and *Desulfovibrio* spp., and also reestablished the Bacteroidetes/Firmicutes ratio (203).

De Angelis et al. address the issue using the metabolomic approach. In particular, they found that ASD patients have: 1) an increase in fecal free amino acids which correlates with an increase in the gut colonization of proteolytic bacteria such as Clostridia (except for *C. Barletti*) and Bacteroides, 2) an alteration in fecal volatile organic compounds suggestive of intestinal dysbiosis, 3) the presence of peptides (indoles and phenols compounds) that play as pseudo-neurotransmitters and neuromodulators, responsible for a reduction in neuroplasticity development and 4) a reduction of SCFAs (such as butyric acid) able to influence neuronal activity and contributing to the so-called “leaky gut” already demonstrated in these patients. The authors also noted an association between the variability of the alterations found and the degree of disease severity: gut dysbiosis is more pronounced in ASD children with significant deficits as compared to pervasive developmental disorder not otherwise specified (209, 210).

ASD involve multiple disabilities with multifactorial etiologies which implies that it must be treated with a holistic approach by a multidisciplinary team. A better understanding of the gut-brain axis in ASD may help clinicians in treating gastrointestinal dysbiosis as soon as possible because this may improve behavioral and cognitive skills. Of course, further studies are needed to understand which specific probiotic or evidence-based nutritional advice should be used according to the patient’s intestinal condition.

#### Parkinson’s Disease

PD is a common degenerative neuromotor disorder affecting 1–2 per 1,000 people worldwide (211). The pathogenesis is multifactorial, and the pathological hallmarks in PD are Lewy body, presence of intraneuronal aggregated alpha-synuclein, and progressive loss of dopaminergic neurons in the substantia nigra compacta. The typical clinical symptoms include bradykinesia, postural instability, and tremor but also gastrointestinal symptoms tract such as constipation and bloating. Moreover, in PD patients, evidence of inflammatory changes has been reported in the brain parenchyma (increased levels of pro-inflammatory cytokines and T cell infiltration) and enteric nervous system.

The gut involvement in PD including increased gut permeability (212) and the presence of gastrointestinal symptoms and inflammation (213) have led to the hypothesis that gut microbiota may have an influence in PD pathogenesis such as in alpha-synuclein aggregation. Recently, an increase in opportunistic pathogens and SCFA-producing bacteria and a decrease in carbohydrate-metabolizing bacteria in PD patients has been demonstrated. The most consistent microbiota changes that can constitute a specific microbial signature of PD are: 1) decrease of Prevotellaceae, 2) increase of Verrucomicrobiaceae and Akkermansia, 3) increased abundance of Bifidobacteria and 4) decreased abundance in Lachnospiraceae (214). Several studies *in vitro* and animal models demonstrated the efficacy of specific probiotic strains in modulating the inflammation in PD.

Magistrelli et al. demonstrated, in an *in vitro* model using PD patients’ PBMCs co-cultured with a selection of probiotic microorganisms belonging to the *Lactobacillus* and *Bifidobacterium* genus, that expression of the pro-inflammatory cytokines IL-1, IL-8, and TNF-α was reduced. In contrast, the expression of the anti-inflammatory regulators TNF-*β* was increased (215). The administration of a probiotic mixture (*Lactobacillus* and *Bifidobacterium*) in two toxin-induced mouse models of PD improved the degeneration of substantia nigra dopaminergic cells and reduced the motor decline by increasing butyrate production, which inhibited nigral inflammation (216).

The main clinical trial on the use of probiotics in PD has been conducted by Tamtaji et al. The authors randomized 60 PD patients to receive either a multistrain probiotic (containing *Lactobacillus* and *Bifidobacterium*) or placebo and demonstrated an improvement in motor signs and symptoms in the probiotic treated group. The clinical effect was associated with higher levels of the antioxidant glutathione and reduced serum levels of C-reactive protein (217).

Pharmacotherapy options for PD are limited at present, and safe non-invasive therapeutic options are needed; this is the reason why probiotics represent an attractive option. However, no solid clinical data are yet available on the real efficacy of this new therapeutic option on motor symptoms and the progression of PD.

## Conclusion

Recently, extensive pieces of evidence are available on local intestinal and systemic immune responses, describing the complicated relationship between foods, bacteria, derived metabolites, and the immune system. The intestinal microbiota, as stated in this review, seems to be primarily involved in the pathogenesis of various ICD characterized by a robust gene–environment interaction. Several bacterial species within the gut are identified as talented players for the onset or maintenance of these conditions. A new approach to ICD will focus on the plethora of factors that may play a role in the vast and still undiscovered world of chronic inflammation that stems from an imbalance of intestinal microbiota: the gut as a door to the body. Restoring what Nature has created and shaped during human evolution and men have changed so dramatically in a couple of centuries is a utopic task. We are now just at the beginning of the understanding of the intimate mechanisms regulating the coevolution of men and microbes. After more than twenty years of research it has appeared clear that each probiotic strain even from the same probiotic species can behave differently according to its specific metabolic pathways, the amount of administered probiotic, the interaction between probiotics and the host, the host itself and its microbiota, the diet, duration of the treatment and all other possible variables. Unfortunately, to have real clinical data that can be replicated and verified, everything needs to be standardized, which is a titanic feat.

Medicine is going toward individualization, and this is also the case of probiotics. In the future, each probiotic treatment will be adapted to the specific patient in that particular clinical situation. Further studies are needed regarding every single aspect of the probiotic therapy, and hopefully, we will reach a robust, patient-tailored biological treatment.

## Author Contributions

All authors contributed to the article and approved the submitted version. All authors take full responsibility for the manuscript.

## Conflict of Interest

RF is the inventor of the patent N 0001425900, released on 17 November 2016 (Italy).

The remaining authors declare that the research was conducted in the absence of any commercial or financial relationships that could be construed as a potential conflict of interest.

## Abbreviations

5-FU, 5-Fluorouracil; 5-ASA, 5-Aminosalicylic acid; ASDs, Autism Spectrum Disorders; EAE, Autoimmune Encephalomyelitis; *B., Bacillus BB., Bifidobacterium* BMI, Body Mass Index; BMDCs, Bone Marrow-Derived Dendritic Cells; CS, Cesarean Section; CeD, Celiac Disease CFS, Cells Free Supernatant; C., Clostridium; CFU, Colony Forming Unit; CD, Crohn’s disease; COX-2, Cyclooxygenase 2; DCs, Dendritic Cells; EcN, *Escherichia coli* strain Nissle 1917; E., *Escherichia*; GFD, Gluten-Free Diet; IgA, Immunoglobulin A; IPA, Indole 3-Propionic Acid; IBD, Inflammatory Bowel Disease; ICD, Inflammatory Chronic Disease; IFN, Interferon; IL, Interleukin; IBS, Irritable Bowel Syndrome; K., Kluyveromyces; LAB, Lactic Acid Bacteria; *L., Lactobacillus*; LPS, Lipopolysaccharides; MAPKs, Mitogen-Activated Protein Kinases; MCP-1, Monocyte chemotactic protein-1; NK, Natural Killer; NEC, Necrotizing Enterocolitis; NOS, Nitric Oxide Synthase; NF-*κ*b, Nuclear Factor kappa-light-chain-enhancer of activated B cells Pathway; PD, Parkinson’s Disease; PRRs, Pattern Recognition Receptors; PBMCs, Peripheral Blood Mononuclear Cells; PSA, Polysaccharide A; PB, Postbiotic; RCT, Randomized Clinical Trial; Breg, Regulatory B Cells; Treg, Regulatory T Cells; *S*., *Saccharomyces*; SFAs, Saturated Fatty Acids; SCFA, Short-Chain Fatty Acid; sVCAM-1, Soluble Vascular Cell Adhesion Molecule 1; Th cells, T helper cells; TLRs, Tool Like Receptors; TNBS, Trinitrobenzoate; TMAO, Trimethylamine N-oxide; TGF, Tumor growth factor; TNF, Tumor Necrosis Factor; UC, Ulcerative colitis; VCAM-1, Vascular Cell Adhesion Molecule 1; WDS, Western-Type Diets.
